# Comparative Cell Biology and Evolution of Annexins in Diplomonads

**DOI:** 10.1128/mSphere.00032-15

**Published:** 2016-03-23

**Authors:** Elin Einarsson, Ásgeir Ástvaldsson, Kjell Hultenby, Jan O. Andersson, Staffan G. Svärd, Jon Jerlström-Hultqvist

**Affiliations:** a Department of Cell and Molecular Biology, BMC, Uppsala University, Uppsala, Sweden; b Department of Laboratory Medicine, Karolinska Institutet, Stockholm, Sweden; Stanford University

**Keywords:** intestinal parasite, annexins, diplomonad, *Spironucleus salmonicida*, *Giardia*, proximity labeling, APEX

## Abstract

Annexins are multifunctional, calcium-binding proteins found in organisms across all kingdoms. Most studies of annexins from single-celled eukaryotes have focused on the alpha-giardins, proteins assigned to the group E annexins, expressed by the diplomonad *Giardia intestinalis*. We have characterized the annexin gene family in another diplomonad parasite, *Spironucleus salmonicida*, by phylogenetic and experimental approaches. We constructed a comprehensive phylogeny of the diplomonad group E annexins and found that they are abundant across the group with frequent gene duplications and losses. The annexins of *S. salmonicida* were found to be related to alpha-giardins but with better-preserved type II Ca^2+^ coordination sites. Two annexins were confirmed to bind phospholipids in a Ca^2+^-dependent fashion but with different specificities. Superresolution and confocal microscopy of epitope-tagged *S. salmonicida* annexins revealed localization to distinct parts of the cytoskeleton and membrane. The ultrastructural details of the localization of several annexins were determined by proximity labeling and transmission electron microscopy. Two annexins localize to a novel cytoskeletal structure in the anterior of the cell. Our results show that the annexin gene family is expanded in diplomonads and that these group E annexins are associated mostly with cytoskeletal and membrane structures.

**IMPORTANCE** Annexins are proteins that associate with phospholipids in a Ca^2+^-dependent fashion. These proteins have been intensely studied in animals and plants because of their importance in diverse cellular processes, yet very little is known about annexins in single-celled eukaryotes, which represent the largest diversity of organisms. The human intestinal parasite *Giardia intestinalis* is known to have more annexins than humans, and they contribute to its pathogenic potential. In this study, we investigated the annexin complement in the salmon pathogen *Spironucleus salmonicida*, a relative of *G. intestinalis*. We found that *S. salmonicida* has a large repertoire of annexins and that the gene family has expanded separately across diplomonads, with members showing sequence diversity similar to that seen across kingdom-level groups such as plants and animals. *S. salmonicida* annexins are prominent components of the cytoskeleton and membrane. Two annexins are associated with a previously unrecognized structure in the anterior of the cell.

## Abstract (precis)

Annexins are proteins that associate with phospholipids in a Ca^2+^-dependent fashion. These proteins have been intensely studied in animals and plants because of their importance in diverse cellular processes, yet very little is known about annexins in single-celled eukaryotes, which represent the largest diversity of organisms. The human intestinal parasite *Giardia intestinalis* is known to have more annexins than humans, and they contribute to its pathogenic potential. In this study, we investigated the annexin complement in the salmon pathogen *Spironucleus salmonicida*, a relative of *G. intestinalis*. We found that *S. salmonicida* has a large repertoire of annexins and that the gene family has expanded separately across diplomonads, with members showing sequence diversity similar to that seen across kingdom-level groups such as plants and animals. *S. salmonicida* annexins are prominent components of the cytoskeleton and membrane. Two annexins are associated with a previously unrecognized structure in the anterior of the cell.

## INTRODUCTION

The annexins are soluble proteins with a distinctive fold, the annexin fold, that bind phospholipids (1). The binding of annexins to acidic phospholipids or other interaction partners can be modulated by the presence of Ca^2+^ ions and influences diverse cellular processes such as vesicle trafficking, signaling, and membrane-cytoskeleton interactions (2).

The annexins have a patchy phylogenetic distribution in eukaryotes, but homologs have been detected in the genomes of animals (group A and B), fungi (group C), plants (group D), and some protists of the Excavata and SAR supergroups (group E) (3). Annexin diversity has been well studied in animals and plants, but comparatively little is known about the evolution, diversity, and characteristics of annexin homologs from protists. The annexin homologs in the diplomonad *Giardia intestinalis*, also designated alpha-giardins, are the best-studied annexins assigned to group E (4). The alpha-giardins were originally discovered as prominent proteins of the structurally complex *Giardia intestinalis* cytoskeleton and found to be immunodominant during human *G. intestinalis* infection (5, 6). Annexins in parasites have been suggested to be good vaccine candidates (7), and immunization with a live, recombinant alpha-1 giardin vaccine formulation provided protection from a *Giardia* challenge in a murine model (8). Three distinct crystal structures of alpha-giardins (alpha-1, -11, and -14 giardins) have been solved, and this has revealed that they contain the typical all alpha-helical annexin fold but with unusual calcium coordination schemes not known in other annexins (9–11). These features give alpha-giardins unique properties; the alpha-1 giardin response to the Ca^2+^ concentration is highly unusual, with abrogated binding to acidic phospholipids at high concentrations, a feature that might be coupled to environmental sensing early during host colonization (11). Sequencing of the first *G. intestinalis* genome revealed the presence of 21 alpha-giardin genes that showed clear evidence of repeated duplications and divergence (4, 12). Annexin homologs from *Spironucleus*, another parasitic diplomonad lineage, were reported to be more similar to other annexins than to alpha-giardins (13). Nothing is known about the biological role of annexins in *Spironucleus*, but recently, the publication of the *Spironucleus salmonicida* genome and the development of an efficient transfection system have enabled comprehensive molecular studies to be performed (14, 15). The large evolutionary distance between *Giardia* and *Spironucleus* and large ultrastructural differences present an interesting system in which to explore the diversity and function of a large gene family spread across several major branches of the eukaryotic tree.

In this study, we took an evolutionary cell biology approach (16) to study the annexin homologs of *S. salmonicida*, combining experimental and phylogenetic methods. We demonstrate the distinct profiles of Ca^2+^ association with membrane lipids of two of the *S. salmonicida* annexins. The annexins constitute expanded gene families in different diplomonad lineages, and the 14 distinct annexins of *S. salmonicida* show subcellular localization to the cytoskeleton, membrane, and cytosol. Proximity labeling with engineered ascorbate peroxidase (APEX) fusions and 3,3ʹ-diaminobenzidine (DAB) as the substrate coupled to transmission electron microscopy (TEM) revealed the fine ultrastructural details of the localization of a subset of the annexins, including two that demarcate a novel cellular structure possibly involved in cell adherence. This study, combined with earlier results, shows that the annexin family is expanded in diplomonads and that many of these group E annexins interact with different cytoskeletal structures or the membrane.

## RESULTS

### Identification of annexin genes in the *S. salmonicida* genome.

The presence of annexin homologs in the draft genome of *S. salmonicida* was investigated by reciprocal BLASTP searches employing alpha-giardins (giardial annexin homologs) and human annexin V as queries. This search identified 16 full-length annexin homologs, 14 of which were encoded by distinct genes (*ANX1* to -*14*; Table 1). Two genes, *ANX3* and -*9*, were each present in two identical copies. The *S. salmonicida* annexins displayed 23 to 41% amino acid sequence identity to alpha-giardins, as well as annexins from nondiplomonad eukaryotes. Only 3 of the 14 *S. salmonicida* annexins showed the highest identity to an alpha-giardin.

**TABLE 1:** Details of *S. salmonicida* annexins

Name	ORF^a^	NCBI accession no.	Amino acid	Mol wt^b^	pI^b^	Extension length (aa)		Expression (FPKM)^c^
						N terminus	C terminus	
ANX1	SS50377_18313	EST42007.1	303	35,620	7.7	7	3 (KAQ)	75.240
ANX2	SS50377_18314	EST42008.1	304	34,579	5.3	7	3 (DAK)	249.132
ANX3	Not annotated	Not annotated	321	35,512	5.1	24	2 (GL)	NA^d^
ANX4	SS50377_12156	EST47757.1	313	36,021	5.2	16	3 (GQE)	612.426
ANX5	SS50377_10477	EST49256.1	311	35,640	5.1	16	2 (GF)	435.131
ANX6	SS50377_12139	EST47740.1	281	32,160	5.5	14	3 (GLK)	8.416
ANX7	SS50377_15726	EST44420.1	309	35,607	7.2	13	4 (NLKS)	7.091
ANX8	SS50377_18312	EST42006.1	299	34,581	5.5	7	1 (N)	2.813
ANX9	SS50377_14057	EST46067.1	300	33,997	6.6	3	3 (GLS)	307.464
ANX10	SS50377_17349	EST43046.1	299	34,021	8.9	6	13 (ENVGG NLFKKC CG)	118.044
ANX11	SS50377_12743	EST47233.1	314	36,141	5.3	13	3 (GFK)	1.395
ANX12	SS50377_12051	EST47860.1	329	36,344	6.2	31	2 (GL)	1,555.338
ANX13	SS50377_15889	EST44286.1	459	53,675	4.7	176	2 (QI)	16.638
ANX14	SS50377_15273	EST44827.1	336	37,960	8.7	2	0	9.756

a ORF, open reading frame.

b Biophysical values predicted by ExPASy ProtParam.

c RNA-Seq expression value from mixed logarithmic and stationary-phase culture extracted from GiardiaDB. FPKM, fragments per kilobase of transcript per million fragments mapped.

d NA, not applicable.

### Primary structure of *S. salmonicida* annexins.

**FIG 1:**
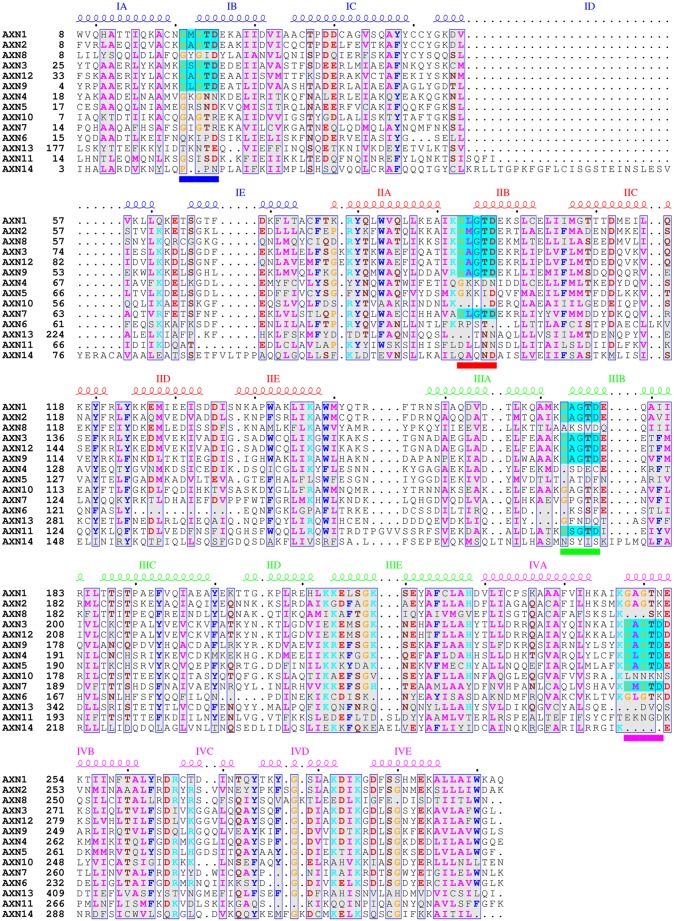
Alignment of *S. salmonicida* annexin sequences showing conserved AB loops. The amino acid sequences of *S. salmonicida* annexins 1 to 14 were aligned by MAFFT (39), and amino acids with physiochemical properties in common are indicated by color if the group similarity is >0.6. Putative type II Ca^2+^ coordination sites (AB loops) matching the G-X-G-T-D/E motif are indicated by cyan shading for each individual occurrence, and the sites in the alignments are underlined. Secondary-structure elements were derived by comparison to the annotated secondary structure of alpha-1 giardin (Protein Data Bank code 4EVF), and the four endonexin repeats (I to IV) and alpha-helices (A to E) are indicated (repeat I is blue, repeat II is red, repeat III is green, and repeat IV is pink). The sequences were truncated at the start of the first endonexin repeat, and the number to the left of each sequence is the amino acid in the alignment. The image shown was prepared with ESPript 3.0 (44).

We performed comparative analyses of *S. salmonicida* annexins to investigate the presence or absence of signature motifs typically found in annexins and/or alpha-giardins. A discriminative motif search of *S. salmonicida* annexins versus *Giardia* alpha-giardins with the MEME tool (45) revealed a motif that is identical or highly similar to AB loops (type II Ca^2+^ coordination sites) in the former. Intact AB loops are unusual in alpha-giardins, with examples found only in alpha-14 giardin (17). An alignment of *S. salmonicida* annexins shows that they have at least one canonical AB loop across the four endonexin repeats (G-X-G-T-D-38X-D/E), except that annexin 13 appears to have lost the type II site in all four domains (Fig. 1). Annexins 3, 9, and 12 have canonical AB loops in all four repeats.

Annexins in animals are known to display divergent N-terminal sequences (before the first annexin repeat) that determine their interactions with other proteins (18). The N-terminal sequences of *S. salmonicida* annexins are mostly short (3 to 176 amino acids [aa]), with 10 extensions being shorter than 18 aa. This unusual feature has previously been observed in alpha-giardins and plant annexins (18). The clear exception is annexin 13, which is substantially larger than the other annexins in *S. salmonicida* and has an additional domain with low similarity to DNA sulfur modification protein DndD, which has been described as a putative structural maintenance of chromosomes protein-like ATPase (19).

The N-terminal domain of vertebrate annexins is known to be modified by phosphorylation, transglutamination, myristoylation, and *S*-glutathionylation (20). Lipid modification is also a factor in the localization of alpha-19 giardin in *G. intestinalis* (21). We employed the GPS-Lipid tool (http://lipid.biocuckoo.org/) (22) to predict potential modification positions in the annexins (see Fig. S1 in the supplemental material). Annexins 3 and 12 were each predicted to have potential palmitoylation sites and one myristoylation site resembling the alpha-19 giardin dual-acylation signal (21). Single and dual sites for geranylgeranyl and farnesylation in the C-terminal region were predicted for annexins 6 and 10, respectively.

10.1128/mSphere.00032-15.1 Figure S1  Predicted lipid modification sites in *S. salmonicida* annexins. The potential for lipid modification was investigated with the GPS-Lipid tool (http://lipid.biocuckoo.org/), which predicts the potential for *N*-myristoylation, *S*-palmitoylation, *S*-farnesylation, and *S*-geranylgeranylation in proteins. A high stringency threshold was employed in the prediction. Download Figure S1, DOCX file, 0.02 MB. Copyright © 2016 Einarsson et al. 2016 Einarsson et al. This content is distributed under the terms of the Creative Commons Attribution 4.0 International license.

To conclude, the primary sequence analyses of *Spironucleus* annexins indicate that, despite being highly divergent, they have retained Ca^2+^ coordination via AB loops. Some members might be targets for protein modifications like lipid acylation to aid in subcellular localization.

### Divergent annexin homologs of *Spironucleus* are *bona fide* annexins.

**FIG 2:**
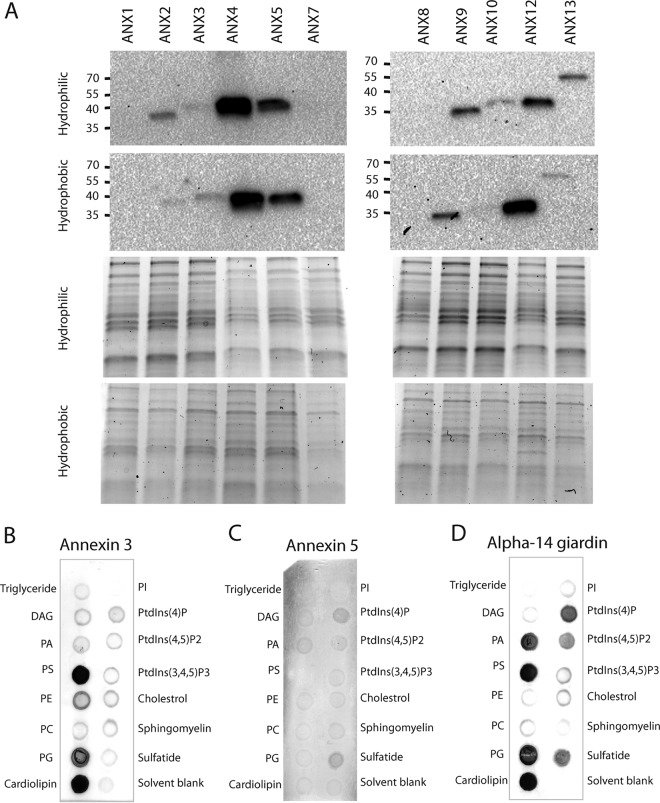
Membrane- and phospholipid-binding characteristics of *S. salmonicida* annexins. Membrane fractionation of transfected cell lines was performed to enrich for integral membrane proteins. (A) The presence of the annexins in the resulting hydrophilic (cytoplasmic) and hydrophobic (membrane) fractions was analyzed by Western blotting with the HA epitope tag. The lower two panel rows show the corresponding loading controls with the Bio-Rad stain-free TGX system. Membrane strips containing 15 biologically active lipids were used to investigate the phospholipid-binding preferences of purified recombinant annexin 3 (B), annexin 5 (C), and alpha-14 giardin (D). Abbreviations: DAG, diacylglycerol; PA, phosphatidic acid; PE, phosphatidylethanolamine; PC, phosphatidylcholine; PI, phosphatidylinositol; PtdIns(3,5)P_3_, phosphatidylinositol 3,5-triphosphate. Molecular sizes in kilodaltons are indicated to the left of the blots.

Many annexins are associated with membrane structures, their binding by definition being regulated by the Ca^2+^ concentration. As a first step to verify that *S. salmonicida* annexin homologs are *bona fide* annexins, we fractionated annexin-expressing transfectant cells to determine if they are associated with the cell membranes. We established transfectant cell lines expressing C-terminally epitope-tagged annexins from their native promoters (except annexin 7, which was expressed from the annexin 3 promoter). The presence of each annexin (except annexins 6, 8, and 11) in the cytosolic and membranous fraction of the transfectants was investigated by detergent-based selective fractionation with the Mem-PER reagent and Western blotting. Annexins 4, 5, 9, and 12 were distributed in both the cytosolic and membrane fractions, while much weaker membrane associations of annexins 2, 3, 10, and 13 were found. Annexin 1 and 8 signals were below the detection level in either fraction (Fig. 2A; see Fig. S2A in the supplemental material).

10.1128/mSphere.00032-15.2 Figure S2  Membrane-binding quantification of *S. salmonicida* annexins. (A) Fractionation of transfected cell line membranes was performed to enrich for integral membrane proteins. The presence of the annexins in the resulting hydrophilic (cytoplasmic, cyt) and hydrophobic (membrane, mem) fractions was analyzed by Western blotting by using the HA epitope tag. The intensities of the bands in the Western blot assay were analyzed by the Quantity One software (Bio-Rad) and are shown in a bar graph. Gray bars mark the cytoplasmic fractions, and black bars mark the membrane fractions. Membrane strips containing 15 biologically active lipids were used to elucidate the binding preference of the purified annexins. Resulting binding patterns for annexin 3 (B), annexin 5 (C), and alpha-14 giardin (D) were quantified by comparing the intensities of bound protein to the background with the Quantity One software and presented in bar graphs. Abbreviations: DAG, diacylglycerol, PA, phosphatidic acid, PE, phosphatidylethanolamine, PC, phosphatidylcholine, PI, phosphatidylinositol, PtdIns(3,4,5)P_3_, phosphatidylinositol 3,4,5-triphosphate. Download Figure S2, DOCX file, 0.2 MB. Copyright © 2016 Einarsson et al. 2016 Einarsson et al. This content is distributed under the terms of the Creative Commons Attribution 4.0 International license.

10.1128/mSphere.00032-15.3 Figure S3  Lipid binding experiments with purified, GST-tagged proteins (annexins 3 and 5 and alpha-14 giardin) were performed as described in Materials and Methods. The GST tag alone was included as a negative control, and the PLC-δ1 PH domain was used as a positive control. Binding assays were performed in the presence or absence (EGTA chelation) of Ca^2+^. The assay was executed in duplicate experiments with optimized protein concentrations in the second experiment, hence the differences in binding intensity and changes in background staining. Annexin 5 displayed an overall weaker association with assayed lipids with positive binding of sulfatide and PtdIns(4)P in the presence of Ca^2+^. Additional binding to cardiolipin was detected in the absence of Ca^2+^. Annexin 3 interacted strongly with PS and cardiolipin and to a lesser extent with PG. All interactions were Ca^2+^ dependent. Calcium-dependent binding of the known interaction partners of alpha-14 giardin, i.e., cardiolipin, PS, PtdIns(4)P, and PtdIns(4,5)P_2_, was detected. Sulfatide and PG were found as additional interacting partners in our experimental setup. Numbered items represent the following lipids: 1, triglyceride; 2, diacylglycerol (DAG); 3, phosphatidic acid (PA); 4, PS, 5, phosphatidylethanolamine (PE); 6, phosphatidylcholine (PC); 7, PG; 8, cardiolipin; 9, phosphatidylinositol (PI); 10, PtdIns(4)P; 11, PtdIns(4,5)P_2_; 12, phosphatidylinositol 3,4,5-triphosphate [PtdIns(3,4,5)P_3_]; 13, cholesterol; 14, sphingomyelin; 15, sulfatide; 16, solvent blank. Download Figure S3, DOCX file, 0.3 MB. Copyright © 2016 Einarsson et al. 2016 Einarsson et al. This content is distributed under the terms of the Creative Commons Attribution 4.0 International license.

Next, we investigated the membrane-binding characteristics of two *S. salmonicida* annexin homologs, one being annexin 3 with four canonical AB loops and the second being annexin 5 with a single intact AB loop in the fourth endonexin repeat. Annexins 3 and 5, expressed as glutathione *S*-transferase (GST) fusions, were purified by glutathione affinity purification and incubated on membrane lipid strips that contain 15 different biological lipids. The membrane lipid strips were queried with an anti-GST antibody conjugated to horseradish peroxidase (HRP), followed by colorimetric detection of bound protein. The assay was performed in duplicate in the presence or absence of Ca^2+^ (by EGTA chelation), and GST alone served as a negative control. We employed alpha-14 giardin and the GST-tagged phospholipase C-δ1 (PLC-δ1) domain as positive controls (10). Annexin 3 was found to associate to cardiolipin and phosphatidylserine (PS), with some minor binding also to phosphatidylglycerol (PG), all in a Ca^2+^-dependent fashion (Fig. 2B; see Fig. S2B and 3 in the supplemental material). Annexin 5 binding was weaker under the conditions assayed, but Ca^2+^-dependent binding to phosphatidylinositol 4-phosphate [PtdIns(4)P] and sulfatide could be seen (Fig. 2C; see Fig. S2C and 3). Alpha-14 giardin bound in a pattern similar to that seen previously by Pathuri and colleagues, with prominent binding to cardiolipin, PS, PtdIns(4)P, and phosphatidylinositol(4,5)biphosphate [PtdIns(4,5)P_2_] (10) (Fig. 2D; see Fig. S2D and 3). Less prominent binding to sulfatide and PG was also observed.

In summary, two divergent *S. salmonicida* annexin homologs were shown to be *bona fide* annexins with distinct phospholipid-binding profiles. Several other annexins were further recovered in the membrane fraction by cell fractionation.

### Phylogeny of annexins.

**FIG 3:**
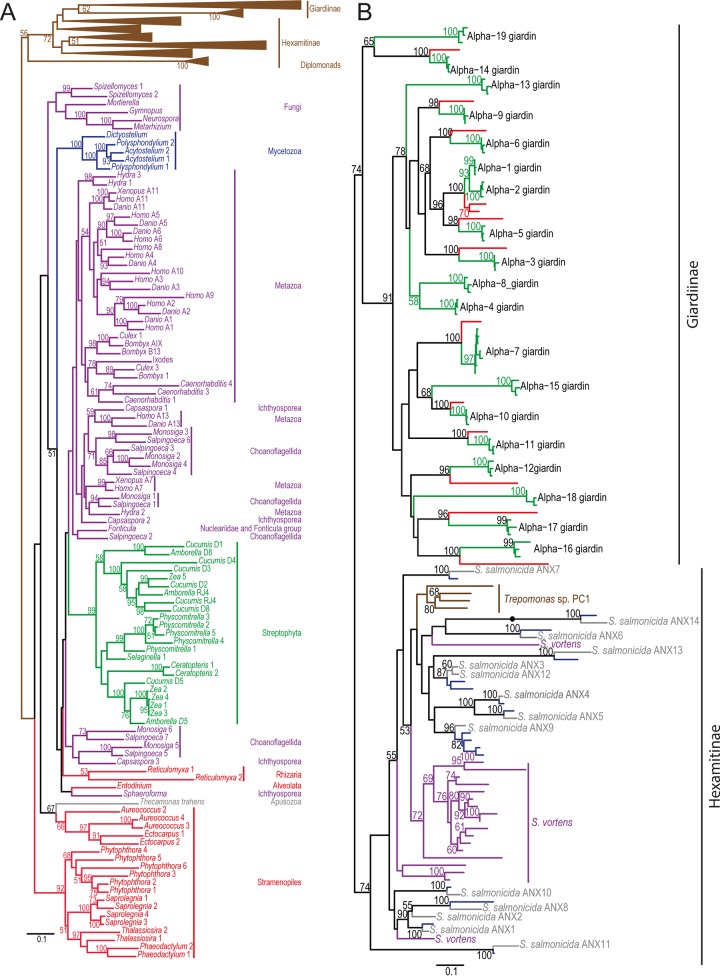
Phylogeny of annexins. Maximum-likelihood phylogenetic analyses of alpha-giardins and annexins from diplomonads and other eukaryotes were performed. (A) Phylogenetic tree based on the whole data set with the diplomonad part of the tree collapsed. The color coding is according to the classification into supergroups as follows (23): SAR, red; *Archaeplastida*, green; *Excavata*, brown; *Amoebozoa*, blue; *Opisthokonta*, purple. (B) Phylogenetic tree based on only the diplomonad sequences. The tree is rooted according to the diplomonad phylogeny, and the root of the diplomonad subtree is indicated by a filled circle. The color coding is according to species as follows: *G. intestinalis*, green; *G. muris*, red; *S. barkhanus*, blue; *S. salmonicida*, gray; *S. vortens*, purple; *Trepomonas* sp. strain PC1, brown. Only bootstrap support values of >50% are shown. For clarity, no bootstrap support values for bipartitions between *G. intestinalis* isolates are shown.

We performed a maximum-likelihood phylogenetic analysis to investigate the evolutionary history of annexin homologs in eukaryotes. We included annexins from *S. salmonicida* and those of other hexamitid flagellates (*S. vortens*, *S. barkhanus*, *Trepomonas* sp. strain PC1), alpha-giardins from *Giardia intestinalis* and *Giardia muris*, and annexin homologs representing the diversity of eukaryotes (Fig. 3). Annexin homologs were found in all of the five currently recognized eukaryotic groups (23), indicating that the protein has a wide distribution in eukaryotes.

All diplomonad sequences form a cluster with strikingly large sequence diversity compared to that of other annexins. The hexamitid flagellate (*S. salmonicida*, *S. barkhanus*, *S. vortens*, and *Trepomonas*) annexins are found in a large cluster with alpha-giardins nested within. However, the position of the root within the diplomonad cluster is likely a result of long-branch attraction; the hexamitid annexins and alpha-giardins are possibly sister clades, indicating that the annexin family has expanded independently in the two groups of diplomonads. Accordingly, there are no orthologous annexins between Giardiinae and Hexamitinae in the phylogeny (Fig. 3). The topology of the alpha-giardins agrees with the previous study of alpha-giardins of *G. intestinalis* (4), except in the position of the alpha-3 and alpha-15 giardins. In almost all cases, the *G. muris* homologs branch together with the *G. intestinalis* sequences for a specific alpha-giardin, suggesting functional conservation. *G. muris* alpha-giardins were present at chromosomal loci similar to those in *G. intestinalis*, but some individual genes were found to be missing or rearranged in the genome, indicative of lineage-specific duplications or gene losses (see Fig. S4A to D in the supplemental material). In a recurring theme, the *S. salmonicida* annexins showed clusters on chromosomes 3, 4, and 6 (see Fig. S5A to D). The clusters on chromosomes 3 and 6 appear to represent loci of gene duplication based on the phylogenetic position of the genes involved (Fig. 3). The closely related species *S. salmonicida* and *S. barkhanus* share homologs for most genes, similar to the situation in *G. intestinalis* and *G. muris*. Across Hexamitinae, there are both orthologs and paralogs indicative of multiple gene duplications and losses (Fig. 3).

10.1128/mSphere.00032-15.4 Figure S4  Arrangements of alpha-giardin genes in the *G. muris* genome. (A) The alpha-giardin homologs of *G. muris* Roberts-Thomson homologs were identified and plotted across the five major unitigs of the genome assembly. Nomenclature is based on the corresponding closest homolog of *G. intestinalis* WB. Alpha-giardins are found on three unitigs with two clusters each on unitigs 807 and 795. One pseudogenized copy of alpha-12 giardin is found on unitig 807. Three full-length alpha-giardin genes are distributed in single copies across the genome, with the rest showing evidence of clustering. (B) A pseudogenized copy of alpha-12 giardin (Ψ alpha-12) and alpha-7.1 giardin are found in a head-to-tail arrangement within 13 kb on unitig 807. (C) Alpha-giardins 9, 1, and 6 are found in a cluster on unitig 807. (D) Two clusters of alpha-giardins (alpha-giardin 10 and 11; alpha-giardin 17 and 16) are found within 8 kb on unitig_795. (E) Two alpha-giardins, alpha-giardin 2 and 5, are found in a cluster on unitig_795. Gene size and spacing between genes are not to scale. Download Figure S4, DOCX file, 0.3 MB. Copyright © 2016 Einarsson et al. 2016 Einarsson et al. This content is distributed under the terms of the Creative Commons Attribution 4.0 International license.

10.1128/mSphere.00032-15.5 Figure S5  Arrangements of annexin genes on the *S. salmonicida* genome. (A) The locations of annexin genes are indicated across the nine chromosomes of *S. salmonicida*. Three clusters of annexin genes are found on three different chromosomes. (B) Annexins 4 and 6 are found in a head-to-tail arrangement within 17 kb on chromosome 4. (C) Annexins 1, 2, and 8 are found within 3 kb on chromosome 6. (D) Six annexin genes, two pairs of which are identical, can be found within 240 kb on chromosome 3. Gene size and spacing between genes are not to scale. Download Figure S5, DOCX file, 0.5 MB. Copyright © 2016 Einarsson et al. 2016 Einarsson et al. This content is distributed under the terms of the Creative Commons Attribution 4.0 International license.

### Expression of *S. salmonicida* annexins.

In our earlier characterization of the *S. salmonicida* genome, we studied gene expression during growth *in vitro* by RNA sequencing (RNA-Seq) (15). This and later analyses showed that all of the 14 annexin genes are expressed in *S. salmonicida* trophozoites (Table 1). Western blotting was used to examine annexin expression at the protein level in stably transfected *S. salmonicida* trophozoites. Most of the annexins investigated (1 to 6, 8, 9, and 11 to 14), except annexins 7 and 10, were clearly detected as discrete proteins species with molecular weights consistent with *in silico* predicted values (see Fig. S6 in the supplemental material). Annexin 10 was detected by Western blotting but migrated as a slightly larger polypeptide than the *in silico* predicted size. The expression of annexin 7 was detected only when the gene was placed under the control of an exogenous promoter (see Fig. S6). The levels of protein expression by Western blotting did not correlate well with levels inferred by RNA-Seq (Table 1).

10.1128/mSphere.00032-15.6 Figure S6  Western blot analysis of 3xHA-tagged annexins**.** Proteins from transfectants expressing annexins 1 to 14 were separated by SDS-PAGE and transferred to a PVDF membrane by electroblotting. The proteins were detected on the basis of the 3xHA tag. Membranes were developed with the ECL Plus detection system and recorded with a ChemiDoc (Bio-Rad). A wild-type sample (SSK wt) was included as a control. Molecular sizes in kilodaltons are indicated. Download Figure S6, DOCX file, 0.1 MB. Copyright © 2016 Einarsson et al. 2016 Einarsson et al. This content is distributed under the terms of the Creative Commons Attribution 4.0 International license.

### Immunolocalization of epitope-tagged annexins.

**FIG 4:**
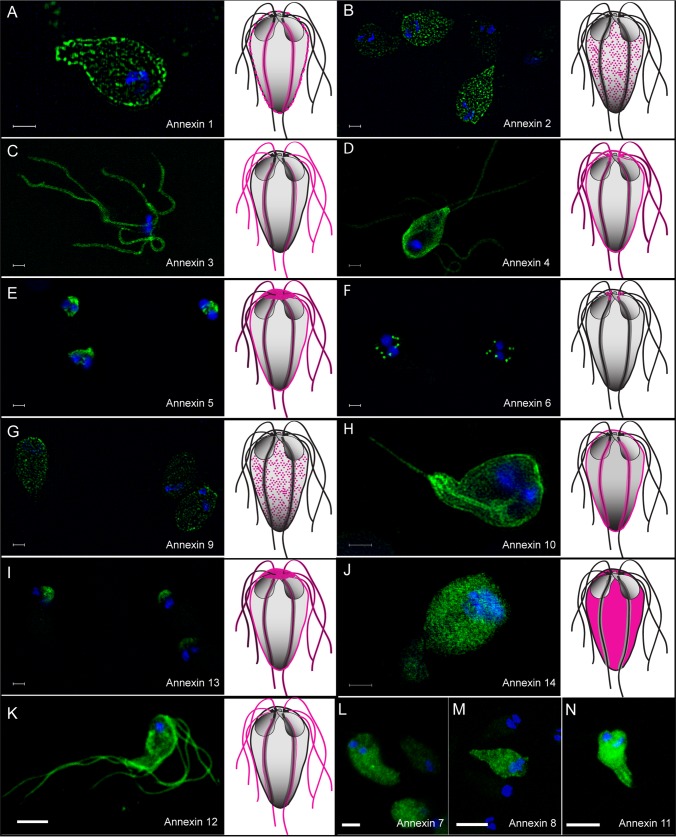
*S. salmonicida* annexin localization. Localization of 3xHA (A, B, D, G, H, and J to N) and V5 (C, E, F, and I) epitope-tagged annexins by superresolution microscopy (A to J) or confocal laser scanning microscopy (K to N). Transfectants were stained with a rabbit anti-HA antibody and detected with an anti-rabbit antibody conjugated to Alexa Fluor 488 (A488) or Alexa Fluor 594 or stained with a mouse anti-V5 antibody and detected with an anti-mouse antibody conjugated to A488. Cells where imaged by a Zeiss LSM510 or LSM710 with a SIM module. Panels: A, annexin 1; B, annexin 2; C, annexin 3; D, annexin 4; E, annexin 5; F, annexin 6; G, annexin 9; H, annexin 10; I, annexin 13; J, annexin 14; K, annexin 12; L, annexin 7; M, annexin 8; N, annexin 11. Scale bars, 2 µm (A to J) and 5 µm (K to N).

In light of the clear association of alpha-giardins with the cytoskeleton in *Giardia* (4), we were interested in obtaining localization data for the *S. salmonicida* annexins. We used the above-described transfectant cell lines expressing C-terminally epitope-tagged constructs of all of the annexins in *S. salmonicida* and imaged them by either confocal laser scanning or structured illumination superresolution microscopy. The tagged annexins displayed a variety of localizations within the cell, and some were very specific in their localization (Fig. 4). Annexin 1 localized to the plasma membrane with a spotty appearance, and significant amounts of label also decorated the internal parts of the recurrent flagella or striated lamina around the recurrent flagella (Fig. 4A). Annexins 2 and 9 were found in numerous cytoplasmic foci (Fig. 4B and G). Annexins 3 and 12 display prominent localization to all flagella, including the internal part of the recurrent flagella (Fig. 4C and K; see Fig. S7 in the supplemental material). The main part of the signal for annexin 4 was present in the plasma membrane, including all eight flagella (Fig. 4D). Annexins 5 and 13 localized to a previously undescribed structure in the anterior part of the cell, and some label was also found in the plasma membrane (Fig. 4E and I). In some cells, a smaller labeled structure is found in the middle portion of the cell body (see Fig. S8). In dividing cells, this smaller structure is translocated to the opposite side of the original structure to the anterior position of the newly forming daughter cell. Annexin 6 uniquely localize to eight, often paired, foci positioned in the anterior part of the cell in most trophozoites (Fig. 4F). Two of the foci are usually located between the nuclei in a position posterior to the other six foci. In cells about to divide, the number of foci is doubled. Annexin 10 seems to be localized to the plasma membrane, but the latter also displayed two fibrous signals that stretch through the cell body along the axis of the recurrent flagella (Fig. 4H). Annexin 7 was not expressed from its own promoter under laboratory conditions, and only upon expression from the annexin 3 promoter could we detect the tagged protein in the cytoplasm (Fig. 4L). Likewise, a mostly cytosolic localization of annexins 8, 11, and 14 was also seen (Fig. 4M, N, and J, respectively).

10.1128/mSphere.00032-15.7 Figure S7  *S. salmonicida* annexins 3 and 12 localize to the flagella. Localization of 3xHA epitope-tagged annexin 3- and 12-expressing *S. salmonicida* cells. Transfectants were stained with rabbit anti-HA and TAT1 antitubulin antibody and detected with an anti-rabbit antibody conjugated to Alexa Fluor 594 (A594) and an anti-mouse antibody conjugated to Alexa Fluor 488 (A488). Cells were viewed in a Zeiss 510 laser scanning confocal microscope. (A) Maximum-intensity projection of A594 (red) and DAPI (blue) in cells expressing epitope-tagged annexin 3. (B) Cells shown in panel A overlaid with TAT1 signal (green). (C) Maximum-intensity projection of A594 (red) and DAPI (blue) in cells expressing epitope-tagged annexin 12. (D) Cells shown in panel C overlaid with TAT1 signal (green). (E) Alignment of the first 20 aa in the N-terminal parts of annexins 3 and 12 and alpha-19 giardin. Putative cysteine acylation sites are indicated by a pink background. Scale bars, 5 µm. Download Figure S7, DOCX file, 1.7 MB. Copyright © 2016 Einarsson et al. 2016 Einarsson et al. This content is distributed under the terms of the Creative Commons Attribution 4.0 International license.

10.1128/mSphere.00032-15.8 Figure S8  *S. salmonicida* annexin 5 localizes at a distinct mid-cell focus in late interphase cells. Localization of V5 epitope-tagged annexin 5 in *S. salmonicida* cells. White arrows show the presence of a second annexin 5 focus appearing in late interphase trophozoites. Transfectants were stained with a mouse anti-V5 antibody and a goat anti-mouse antibody conjugated to Alexa Fluor 488 (A488). In panels A and B, cells were viewed in a Zeiss LSM710 with a SIM module for superresolution. A488 is green, and DAPI is blue. (C) Cell viewed with a Zeiss Axioplan 2 fluorescence microscope. A488 is green. (D) Cartoon of annexin 5 localization in late interphase cells. Scale bars, 2 µm. Download Figure S8, DOCX file, 0.5 MB. Copyright © 2016 Einarsson et al. 2016 Einarsson et al. This content is distributed under the terms of the Creative Commons Attribution 4.0 International license.

### Ultrastructural localization of annexins with APEX.

The function of annexins could be easier to understand if they could be coupled to specific ultrastructural elements in the cell. The possibilities of using genetically encoded proximity labeling tags with broad functionality have recently been realized in several variants (24, 25). We have adapted the APEX system (25) for use in diplomonads with DAB as a substrate to reveal the ultrastructural localization of a fusion partner. We constructed C-terminal APEX-V5 fusions of annexins 3 to 6, 10, and 13 to try to obtain a transect of the diverse set of localizations seen by immunolocalization (Fig. 4). We were unable to generate annexin 4 transfectants expressing the APEX-V5 fusion. For the remaining constructs, we studied the subcellular localization by immunofluorescent staining with the V5 epitope. Annexins 3, 5, 6, and 13 showed localizations identical to those of the hemagglutinin (HA)-tagged constructs (Fig. 4). However, annexin 10 was only sporadically expressed and cells with expression did not show localization to the recurrent-flagellar region as in the HA-tagged construct.

**FIG 5:**
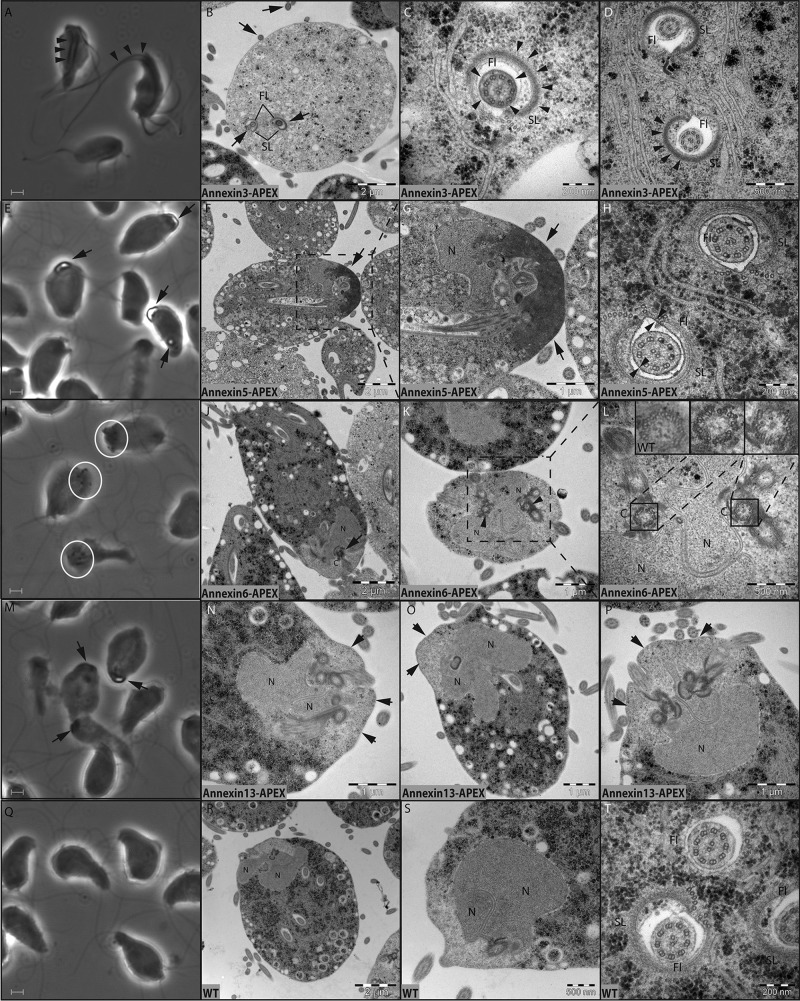
Ultrastructural details of annexin localization through APEX-based proximity labeling. Transfectants expressing annexins fused to a C-terminal APEX-V5 cassette were reacted with DAB and then subjected to OsO_4_ treatment. The fixed cells were encased in resin, and thin slices were prepared for TEM. Arrows and circled areas in the micrographs indicate sites of DAB precipitates shown as regions of increased contrast compared to DAB-stained wild-type cells. (A, E, I, M, and Q) Cells were viewed by phase-contrast microscopy at ×100 magnification after DAB treatment. Panels: A to D, annexin 3; E to H, annexin 5; I to L, annexin 6; M to P, annexin 13; Q to T, DAB-stained wild-type cells. Abbreviations: N, nucleus; fl, flagellum; C, centriole; SL, striated lamina. Scale bars in panels A, E, I, M, and Q, 2 µm. Scale bar sizes in TEM images are indicated in the panels.

Transfectant cells grown in hemin-supplemented LYI (liver digest, yeast extract, and iron) growth medium were washed and promptly fixed in glutaraldehyde in cacodylate buffer and reacted with DAB. At that stage, cells were viewed by phase-contrast microscopy (Fig. 5A, E, I, M, and Q). Annexin 3 displayed prominent dark flagella, with the internal part of the recurrent flagella clearly observable (Fig. 5A). Annexin 5 showed a strong curving label in the anterior part of the cell (Fig. 5E). Cells in the stages leading to cell division displayed a smaller label situated in the middle of the cell that progressively seemed to move closer to the anterior as cells grew in size before cell division (Fig. 5E). Annexin 6 exhibited six dark foci in the anterior of the cell, with cells close to cell division showing double the number of foci (Fig. 5I). The foci were often arranged in pairs. Annexin 13 showed labeling similar to that of annexin 5 but with much less intense staining (Fig. 5M).

Light microscopy revealed many details of the subcellular localization of the four annexins investigated, but we were interested in learning the ultrastructural fine details of their localization. Samples were reacted with osmium to make DAB precipitates visible and then prepared for TEM. For annexin 3, we observed increased contrast in the flagella, especially around the outer microtubule doublets of the 9 + 2 axoneme (Fig. 5B to D; see Fig. S9A and B). Biradial staining was also noted in the striated lamina running the length of the recurrent flagella (Fig. 5B to D; see Fig. S9A and C). The annexin 5 transfectants exhibited strong staining in a large area in the anterior part of the cells (Fig. 5F and G; see Fig. S9D to F). DAB precipitates were also noted on either side of the cell membrane in the flagella, as well as the cell body (Fig. 5H). TEM of annexin 6-APEX revealed a signal in the vicinity of the basal bodies with prominent DAB staining in and around the centrioles (Fig. 5J to L). Subtle DAB staining of annexin 13-APEX was observed in the anterior of the cell in a location similar to that of annexin 5 (Fig. 5N to P). Wild-type cells reacted with DAB showed a staining pattern similar to that of control cells where DAB had been omitted (Fig. 5Q to T). On some occasions, a DAB reaction product could be seen in the interior of microtubules.

10.1128/mSphere.00032-15.9 Figure S9  TEM of proximity-labeled annexin-APEX transfectants reveals ultrastructural details. Transfectants expressing annexins fused to a C-terminal APEX-V5 cassette were fixed, reacted with DAB, and then treated with OsO_4_. The fixed cells were encased in resin, and thin slices were prepared for TEM. Arrows in the micrographs indicate sites of DAB precipitates. Panels: A to C, annexin 3; D to F, annexin 5. Abbreviations: N, nucleus; fl, flagellum; C, centriole; SL, striated lamina; H, hydrogenosome. Scale bar sizes are indicated. Download Figure S9, DOCX file, 0.6 MB. Copyright © 2016 Einarsson et al. 2016 Einarsson et al. This content is distributed under the terms of the Creative Commons Attribution 4.0 International license.

## DISCUSSION

In this study, we investigated members of the annexin E gene family in diplomonads, with an emphasis on *S. salmonicida*, by both phylogenetic analyses and experimental approaches. We also identified alpha-giardins from a second *Giardia* species, *Giardia muris*. The partition between alpha-giardins and annexins from *Spironucleus* and *Trepomonas* in the phylogenetic analysis suggests that either a single annexin gene was present in the ancestral diplomonad or the family has a high rate of gene turnover within diplomonads. The diversity of the annexin sequences observed in diplomonads is similar to, or even larger than, the diversity of annexin sequences from different eukaryotic supergroups (Fig. 3). It would be very interesting to investigate the occurrence of annexins in free-living relatives of diplomonads, especially since no annexin homologs from other members of the Excavata supergroup are known. The human genome encodes 12 annexins, whereas *G. intestinalis* and *S. salmonicida* have 19 and 14 different annexins, respectively. It is still not known why the annexins have been expanded in the diplomonads, but it is tempting to speculate that the less complex actin cytoskeletons of parasitic diplomonads have led to the expansion of annexins to replace this loss (26).

The *Spironucleus* annexins appear to be less derived than alpha-giardins, and most harbor at least one type II Ca^2+^ coordination site. In accordance with this observation, we showed Ca^2+^-dependent interaction between two *S. salmonicida* annexins and acidic phospholipids, establishing that they are *bona fide* annexins. Moreover, the annexins displayed different binding preferences, in line with their low sequence identity and nonoverlapping cellular localizations. There might be a correlation between the number of intact type II sites and the strength of membrane association since annexins 3, 9, and 12 all have four canonical sites and are found in the membrane fraction. At the same time, the annexin with the most prominent membrane association, annexin 4, has only a single conserved repeat and presumably associates by a separate mechanism. Several annexins showed a potential for posttranslational modifications via acylation, a property previously described in the N-terminal alpha-19 giardin (21). Consequently, annexins 3 and 12 also localize to the flagellum, like alpha-19 giardin (see Fig. S7 in the supplemental material). The C terminus of annexin 10 carries an extension of 14 aa with two paired cysteine residues in the second- and third-last residues and shows a slight shift in molecular size from calculated values during SDS-PAGE (Table 1; see Fig. S6 in the supplemental material). The large APEX-V5 tag led to mislocalization of annexin 10, while the small 3xHA epitope tag appears to be tolerated. We speculate that this phenotype is due to the inability of annexin 10 to become acylated and attain its intended cellular location. Unfortunately, without TEM data, we were unable to determine if annexin 10 localizes to the recurrent flagella, internal membranes, or striated lamina (27).

Many of the strongly expressed annexins in *S. salmonicida* localize to the membrane or cytoskeleton, while more weakly expressed annexins often localize to the cytosol (Fig. 4). It is possible that these weakly expressed annexins play a more prominent role during other parts of the poorly described *S. salmonicida* life cycle. Two of the annexins, 5 and 13, were found to localize to a previously undescribed cellular organelle in the anterior end of the cell above the nuclei (Fig. 4E and I). The structure labeled by these two proteins is homogeneous and electrolucent in TEM of wild-type cells and corresponds to the region of the cell that appears to mediate attachment to various substrates. In the related flagellate *Spironucleus torosa*, which infects gadoid fish, this area of the cell has been seen to mediate intimate contact with the epithelium of the host (28). The *S. salmonicida* genome lacks signature proteins (beta-, delta-, and gamma-giardins; SALP-1; median body protein) of the *Giardia* ventral disc. On the other hand, at least three alpha-giardins have been found associated with this attachment organelle (29). It is possible that *S. salmonicida* has evolved an attachment organelle by co-opting homologous proteins such as those found in the *Giardia* ventral disc.

The localization of annexin 3 was also studied by APEX proximity labeling. Annexin 3 localizes prominently to the flagella, and DAB staining also labels the striated lamina that supports the internal portion of the recurrent flagella. Annexin 3 interacted with cardiolipin, PS, and PG, the first two of which are also binding partners of alpha-14 giardin, which is found in giardial flagella (10). Besides membrane interaction, alpha-14 giardin is known to interact with tubulin, has the capacity to self-assemble into multimeric structures, and forms local slubs in the flagella at the proximal and distal ends of the axoneme (30). Analogously, annexins 3 and 12 colocalize with tubulin in *S. salmonicida* by confocal microscopy, and proximity labeling suggests a close association with tubulin (see Fig. S7 in the supplemental material). Superresolution microscopy revealed that annexin 3 is also unevenly distributed in the flagella, reminiscent of the slubs observed in *Giardia* (30). The development of proximity labeling techniques using the APEX peroxidase and other substrates offers a promising avenue by which to investigate interaction partners. Future studies will be directed at unraveling the makeup of the novel anterior organelle and its putative role in attachment. Additionally, we will attempt to find interaction partners of annexins in *S. salmonicida* to learn more about their cellular roles.

The alpha-giardins in *Giardia* were the first annexins from a protozoan eukaryote to be described (31–33). Our study and previous studies of alpha-giardins (4, 6, 17, 21, 30, 32, 34, 35) have provided insights into the functional roles of members of the annexin protein family in diplomonads in particular and eukaryotic cells in general. Furthermore, our bioinformatic search identified annexin homologs from a wide range of microbial eukaryotes representing all five major groups (Fig. 3) (23). Most of these are unicellular and should probably be classified as group E annexins according to the current annexin nomenclature (36). This is problematic since unicellular eukaryotes are not a monophyletic group and many of the identified annexins of protists (Fig. 3) are more closely related to, and probably share more functional features with, annexins in the A, B, C, or D group than the group E annexins studied here. We think that the annexin classification system needs to be reevaluated in light of the current knowledge of annexin distribution in eukaryotes and eukaryote phylogeny.

The phylogenetic analysis suggests that the annexin protein family probably was present early in eukaryote evolution (Fig. 3). However, annexins are not specific for eukaryotes. Thirty-four putative bacterial annexin proteins have been identified in 17 different bacterial species (37). Unfortunately, the bacterial annexin domains are divergent from the eukaryotic annexins and do not show affinity to any specific eukaryotic group. Therefore, the evolutionary origin of annexins in eukaryotes is impossible to pinpoint on the basis of the current data.

Additional studies of annexins from diverse protists would provide a deeper understanding of different functions of annexins. For example, annexin homologs are present in unicellular sister groups of animals such as *Choanoflagellida* and *Ichthyosporea*. Phylogenetic analysis (Fig. 3) suggests that multiple paralogs of annexins were indeed present in the last common ancestor of these protist groups and animals. Functional studies of annexins of these protists, similar to our study, would be very useful to understand the function of annexins of animals and humans. We believe that a combination of genomic and phylogenetic analyses and experimental approaches such as those used here in studies of new microbes from different parts of the tree of life will further reveal the function and evolutionary history of annexins in eukaryotes.

## MATERIALS AND METHODS

### Cell culture.

*S. salmonicida* (ATCC 50377, previously ATCC 50380), was cultured at 16°C in slanted polypropylene tubes (Nunc) in modified LYI culture medium as described previously (14).

### Epitope tagging vector construction and transfection.

The coding sequences and 100 to 400 bp of the putative promoters of the 14 annexin genes were PCR amplified from genomic DNA of *S. salmonicida* (see Table S1 in the supplemental material). The PCR products were column purified, digested with appropriate restriction enzymes, and ligated into vector pSpiro-PAC-3xHA-C (14) or pSpiro-PAC-APEX-V5. The pSpiro-PAC-APEX-V5 vector was created by amplifying the pea APEX gene from pcDNA3 Connexin43-APEX (Addgene plasmid 44439) (25) with the APEX-F and APEX-V5-R primers and inserting it in place of the 3xHA tag at the NotI and ApaI sites of pSpiro-PAC-3xHA-C. The finished constructs were sequenced to confirm the sequence of the final construct. Transfection of *S. salmonicida* followed the method described previously (14).

10.1128/mSphere.00032-15.10 Table S1  Sequences of primers used for *S. salmonicida* annexin cloning and pSpiro-PAC-APEX-V5 vector construction. Download Table S1, DOCX file, 0.02 MB. Copyright © 2016 Einarsson et al. 2016 Einarsson et al. This content is distributed under the terms of the Creative Commons Attribution 4.0 International license.

### Mem-PER extraction.

Cells were membrane fractionated with the Mem-PER Eukaryotic Membrane protein extraction kit (Pierce 89826) by using the protocol for mammalian cells. Detergents were removed with the SDS-PAGE sample preparation kit (Thermo Scientific 89888) according to the manufacturer’s recommendation. The protein concentrations were measured and normalized with Qubit. SDS-PAGE and Western blotting were performed as described below. Loading controls were generated with the TGX stain-free system (Bio-Rad) according to the manufacturer’s recommendation.

### Protein purification and membrane binding.

Annexins 3 and 5 (resynthesized in standard code), alpha-14 giardin, and GST (empty vector) were cloned into the pGEX-6P-3 vector (GE Healthcare) (38). Proteins were expressed in *Escherichia coli* strain BL21 and purified by binding to glutathione Sepharose 4B (GE Healthcare) according to the manufacturer’s instructions. Eluted proteins were buffer exchanged (10 mM Tris-HCl, 150 mM NaCl, pH 8), concentrated with Vivaspin-6 (molecular mass cutoff, 20 kDa; GE Healthcare), and stored at −20°C.

The lipid-binding capacities of annexins 3 and 5, alpha-14 giardin, and GST were tested with membrane lipid strips (P-6002; Echelon Research Laboratories, Salt Lake City, UT). The membranes were blocked with 3% fatty-acid-free bovine serum albumin (A8806; Sigma) in phosphate-buffered saline–0.1% Tween 20 (pH 7.4) for 1 h. GST-tagged proteins (0.5 µg/ml annexin 3 and alpha-14 giardin, 2 µg/ml annexin 5 and GST) in blocking buffer were incubated with the membrane for 1 h. The PI(4,5)P_2_ Grip binding protein (G-4501; Echelon Research Laboratories, Salt Lake City, UT), derived from the N-terminally GST-tagged recombinant PLC-δ1 PH domain, was used as a positive control and diluted to 0.5 µg/ml according to the manufacturer’s instructions. The membranes were incubated for 1 h with an HRP-coupled anti-GST antibody (K-SEC2; Echelon Research Laboratories, Salt Lake City, UT), and the bound protein was detected with the K-TMBP substrate (Echelon Research Laboratories, Salt Lake City, UT). Lipid-protein interactions were quantified by measuring the intensity of color compared to global background staining with the Quantity One v 4.6.6 software (Bio-Rad).

### Western blotting.

Whole-cell sample preparation for Western blotting and SDS-PAGE is described in reference 14. Proteins were separated with precast polyacrylamide gels (Any kD TGX stain-free gels; Bio-Rad) and transferred to polyvinylidene difluoride (PVDF) membranes. The HA tag was detected with rat anti-HA high-affinity monoclonal clone 3F10 (1:1500; Roche product no. 11867423001), and detection was performed with an HRP-coupled goat anti-rat antibody (1:10,000; Thermo Scientific product no. 31470). The blots were developed with the Clarity Western ECL substrate (Bio-Rad) and recorded on a ChemiDoc MP+ imaging system (Bio-Rad).

### Phylogenetic analyses.

The data set for phylogenetic analyses consisted of the following published and unpublished diplomonad amino acid sequences: 14 full-length annexin sequences from *S. salmonicida*; 21, 20, and 20 alpha-giardins from the *G. intestinalis* WB, GS, and P15 isolate genomes, respectively; 13 alpha-giardins from the ongoing unfinished *G. muris* Roberts-Thomson genome project; 4 annexin homologs from transcriptome data of the free-living diplomonad *Trepomonas* sp. strain PC1; and 19 and 16 annexin homologs from the ongoing unfinished genome projects of *S. vortens* and *S. barkhanus*, respectively. The 127 diplomonad sequences were complemented with 109 sequences selected to represent the diversity of eukaryotes. The data set was aligned with MAFFT v7.215 (39) by using the L-INS-I option. Sites to include in the phylogenetic analysis were manually selected, and RAxML v8.1.15 (40) was used for the phylogenetic analysis with the setting -m PROTGAMMALG4X -f a -n 100. Separate phylogenetic analyses were performed with and without nondiplomonad sequences.

### Immunofluorescence microscopy.

Cells were prepared for immunofluorescence microscopy according to reference 14. The V5 epitope was detected with mouse anti-V5 monoclonal antibody SV5-Pk1 (1:750; AB27671; Abcam). The primary antibody was detected with an Alexa Fluor 488-conjugated goat anti-mouse polyclonal antibody (1:800; A11029; Life Technologies). Tubulin was labeled with a mouse anti-tubulin TAT1 monoclonal antibody (1:150) (41). The primary antibodies were detected with an Alexa Fluor 488-conjugated goat anti-mouse polyclonal antibody (1:250; A-11001; Life Technologies) or an Alexa Fluor 594-conjugated goat anti-rabbit antibody (1:250; catalog no. A-11037; Life Technologies). The cells were viewed with a Zeiss Axioplan 2 fluorescence microscope or a Zeiss 510 laser scanning confocal microscope. The images were processed with the Zen 2011 v7.0.0.285 software (Carl Zeiss GmbH) or the BioImageXD v1.0 RC3 software (42).

### SIM.

Transfected cells were prepared in the same way as for immunofluorescence. Cells were imaged with a Zeiss LSM710 with a structured illumination microscopy (SIM) module for superresolution. The images were processed with the Zen 2012 software (blue edition), ImageJ Fiji (43), and Adobe Illustrator.

### APEX proximity labeling and TEM.

Cells were grown in LYI medium supplemented with 100 µM hemin (H9039; Sigma-Aldrich). Cells were fixed, DAB labeled, and processed for TEM as described in reference 25. The pellets were then postfixed in 2% osmium tetroxide in 0.1 M phosphate buffer, pH 7.4, at 4°C for 2 h; dehydrated in ethanol, followed by acetone; and embedded in LX-112 (Ladd, Burlington, Vermont). Ultrathin sections (approximately 50 to 60 nm) were cut by a Leica Ultracut UCT ultramicrotome (Leica, Vienna, Austria) and examined in a Hitachi HT 7700 (Hitachi, Tokyo, Japan) at 80 kV. Digital images were taken with a Veleta camera (Olympus Soft Imaging Solutions, GmbH, Münster, Germany).

### Nucleotide sequence accession numbers.

DNA sequences of annexin genes from *G. muris*, *S. barkhanus*, and *S. vortens* were deposited at NCBI GenBank under accession numbers KU341410 to KU341457. Annexin sequences for *Trepomonas* sp. strain PC1 have been deposited as a transcriptome shotgun assembly project at DDBJ/EMBL/GenBank under accession number GDID00000000.
