# Supplementary material for: Comparative Cell Biology and Evolution of Annexins in Diplomonads
Source: mSphere. 2016 Mar 23;1(2):e00032-15. doi: 10.1128/mSphere.00032-15 (PMC4863580; doi:10.1128/mSphere.00032-15)
Supplement: Figure S1 [file sph002162052sf1.docx]

| **ID** | **Position** | **Peptide** | **Score** | **Cutoff** | **Type** |
| --- | --- | --- | --- | --- | --- |
| ANX2 | 150 | RLIKAWMCQTRFDRN | 1.413 | 1.396 | S-Palmitoylation: Cluster C |
| ANX3 | 2 | ******MGCSADQKV | 11.755 | 9.218 | N-Myristoylation: Non-consensus |
| ANX3 | 3 | *****MGCSADQKVV | 3.409 | 1.396 | S-Palmitoylation: Cluster C |
| ANX3 | 72 | FNKQYSKCMIESLKK | 3.114 | 3.076 | S-Palmitoylation: Cluster B |
| ANX6 | 273 | LSGDYERCILAVWGL | 9.436 | 6.806 | S-Farnesylation: CAAX |
| ANX6 | 273 | LSGDYERCILAVWGL | 3.143 | 3.059 | S-Geranylgeranylation: CAAX |
| ANX7 | 92 | RYQLWAECIHHAVAG | 4.446 | 3.076 | S-Palmitoylation: Cluster B |
| ANX10 | 305 | GGNLFKKCCG***** | 8.968 | 4.003 | S-Farnesylation: Non-consensus |
| ANX10 | 305 | GGNLFKKCCG***** | 9.442 | 0.486 | S-Geranylgeranylation: CC/CXC |
| ANX10 | 306 | GNLFKKCCG****** | 10.327 | 4.003 | S-Farnesylation: Non-consensus |
| ANX10 | 306 | GNLFKKCCG****** | 2.252 | 0.486 | S-Geranylgeranylation: CC/CXC |
| ANX12 | 2 | ******MGAGSTTSK | 6.317 | 3.173 | N-Myristoylation: MGXXXS/T |
| ANX12 | 13 | TTSKCDKCGHSIKIE | 15.112 | 1.983 | S-Palmitoylation: Cluster A |
| ANX13 | 454 | HDMVDVICSILQI** | 6.957 | 4.003 | S-Farnesylation: Non-consensus |
| ANX13 | 454 | HDMVDVICSILQI** | 4.917 | 1.617 | S-Geranylgeranylation: Non-consensus |
| ANX14 | 38 | HSQVQQLCRAFQQQT | 4.856 | 3.076 | S-Palmitoylation: Cluster B |
| ANX14 | 48 | FQQQTGYCLKRLLTG | 2.307 | 1.983 | S-Palmitoylation: Cluster A |
| ANX14 | 62 | GPKFGFLCISGSTEI | 3.404 | 3.076 | S-Palmitoylation: Cluster B |
| ANX14 | 326 | KELKSQSCGIFKKAI | 10.454 | 4.003 | S-Farnesylation: Non-consensus |
| ANX14 | 326 | KELKSQSCGIFKKAI | 3.768 | 1.617 | S-Geranylgeranylation: Non-consensus |

**Figure S1**
