# Supplementary figures and images for: Comparative Cell Biology and Evolution of Annexins in Diplomonads
Source: mSphere. 2016 Mar 23;1(2):e00032-15. doi: 10.1128/mSphere.00032-15 (PMC4863580; doi:10.1128/mSphere.00032-15)

**
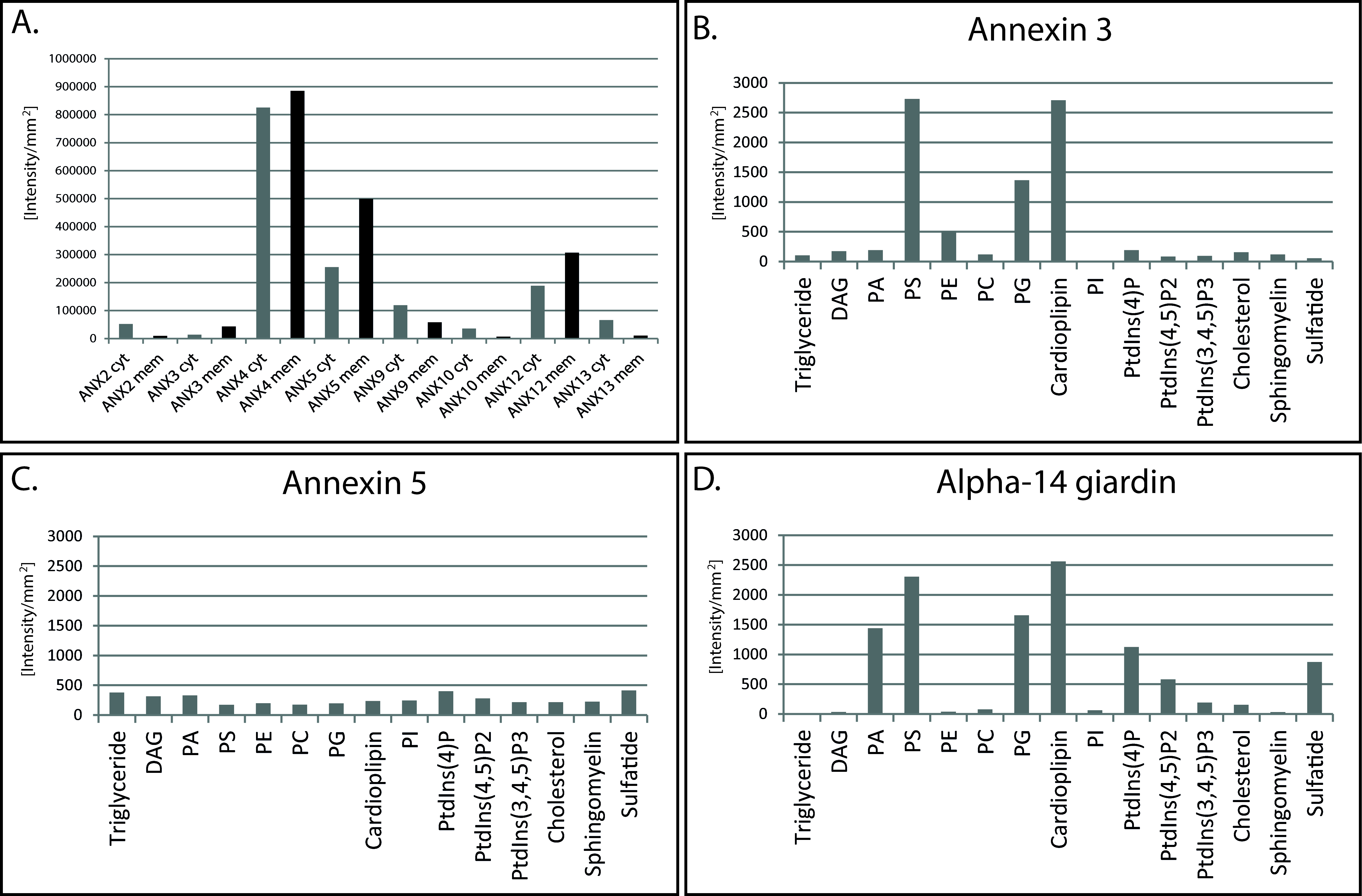
**

**Figure S2**

Supplement: Figure S2 [file sph002162052sf2.docx]

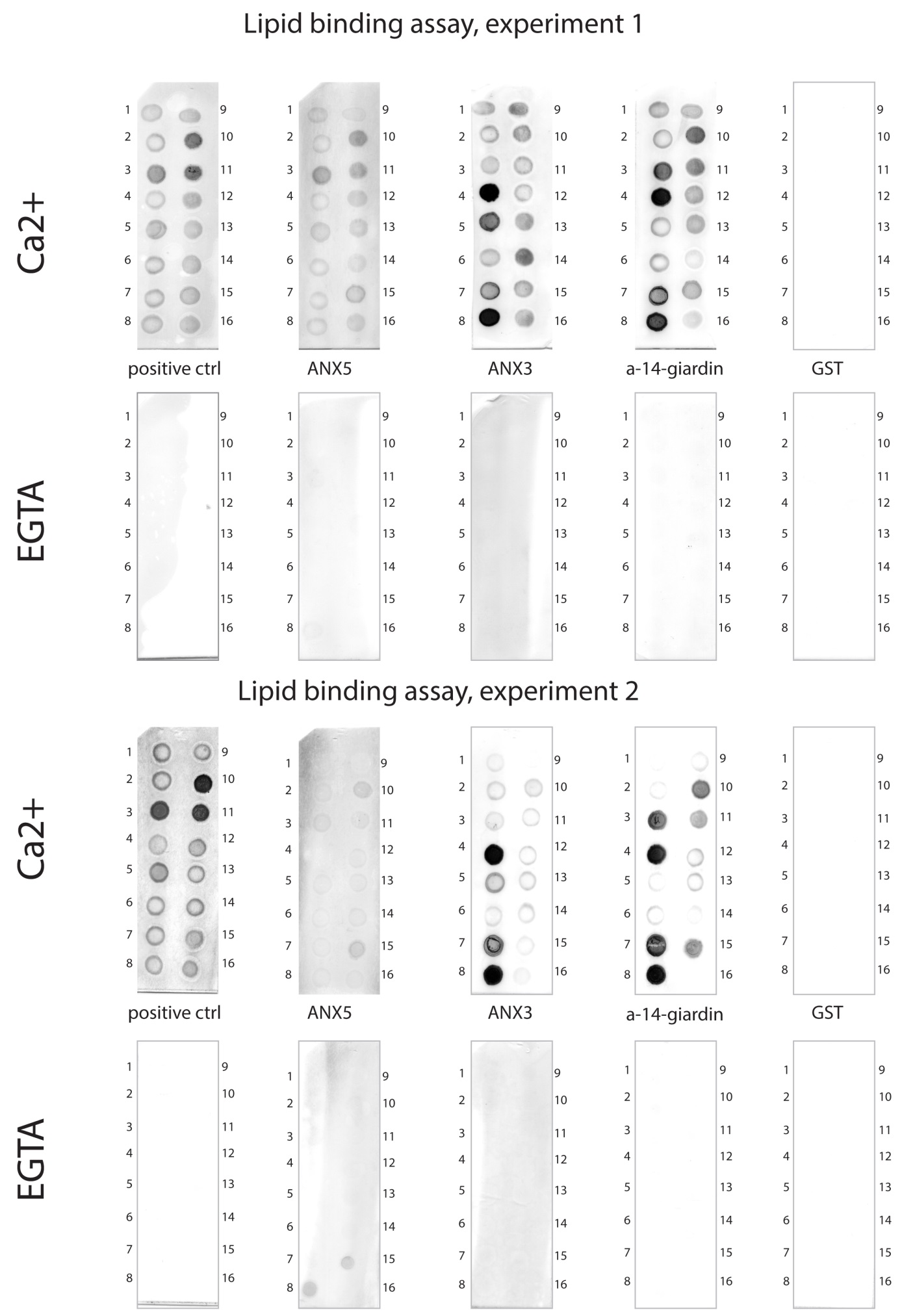


# **Figure S3**

Supplement: Figure S3 [file sph002162052sf3.docx]

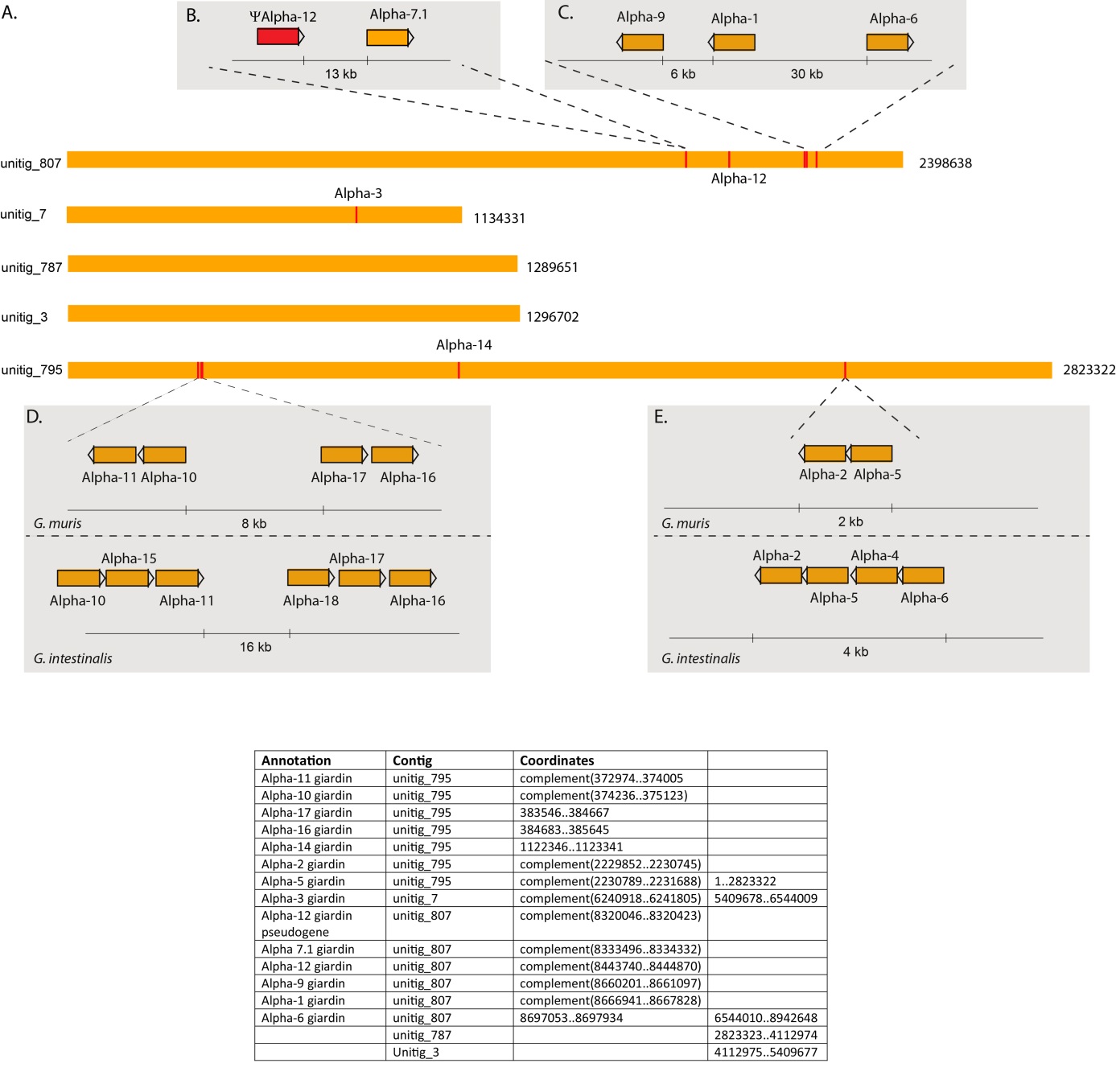


**Figure S4**

Supplement: Figure S4 [file sph002162052sf4.docx]

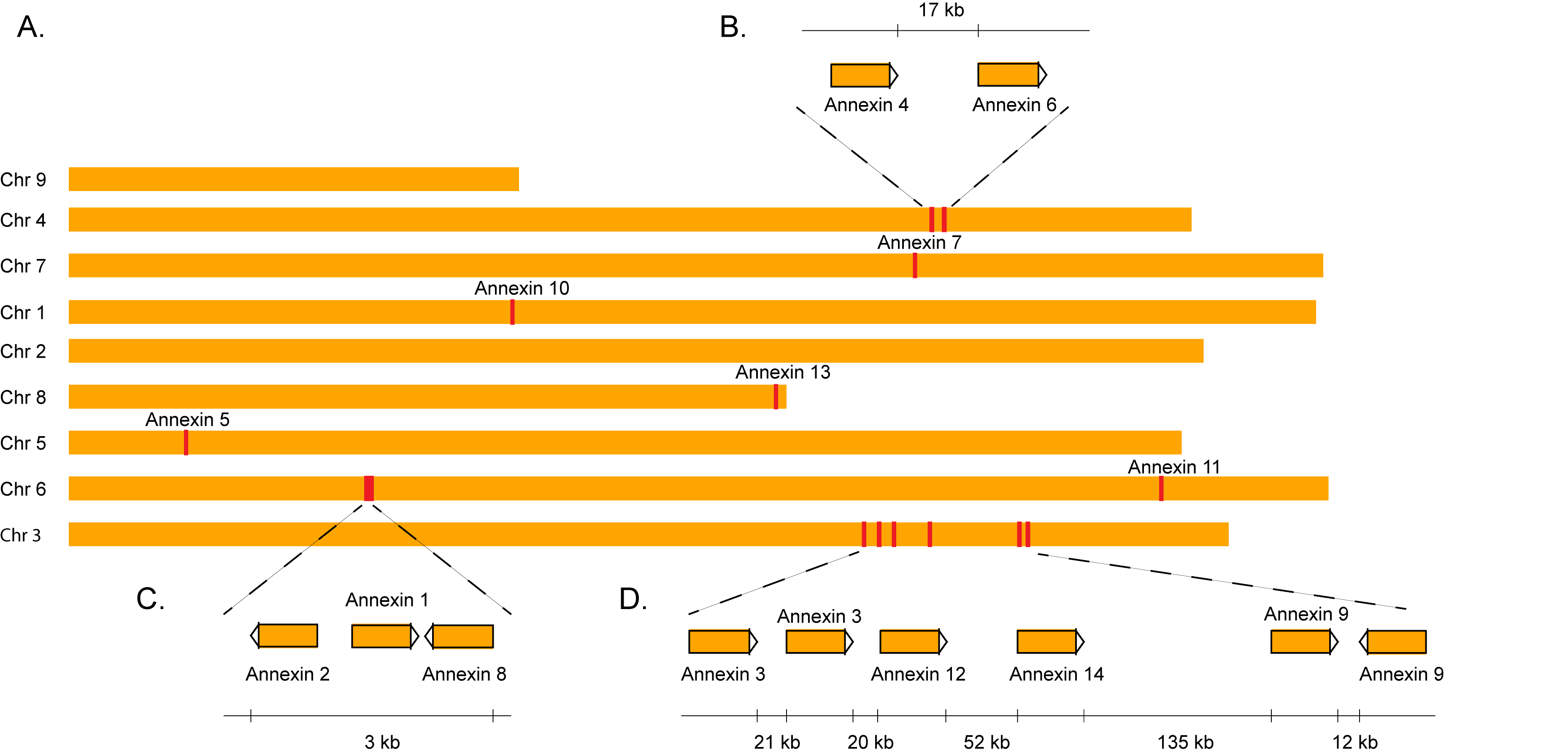


**Figure S5**

Supplement: Figure S5 [file sph002162052sf5.docx]

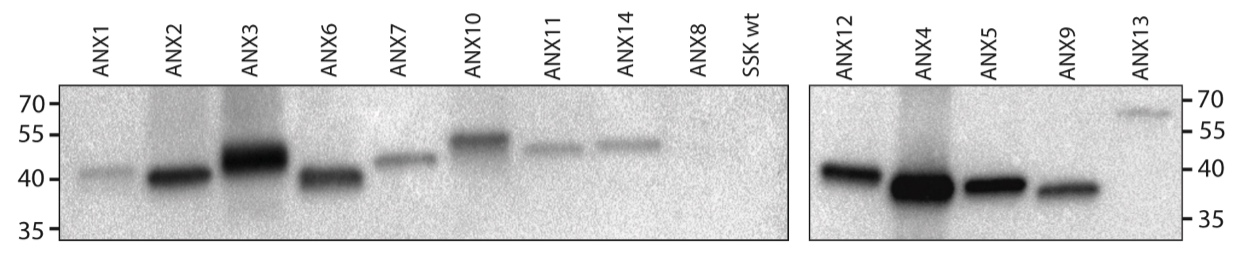


Figure S6

Supplement: Figure S6 [file sph002162052sf6.docx]

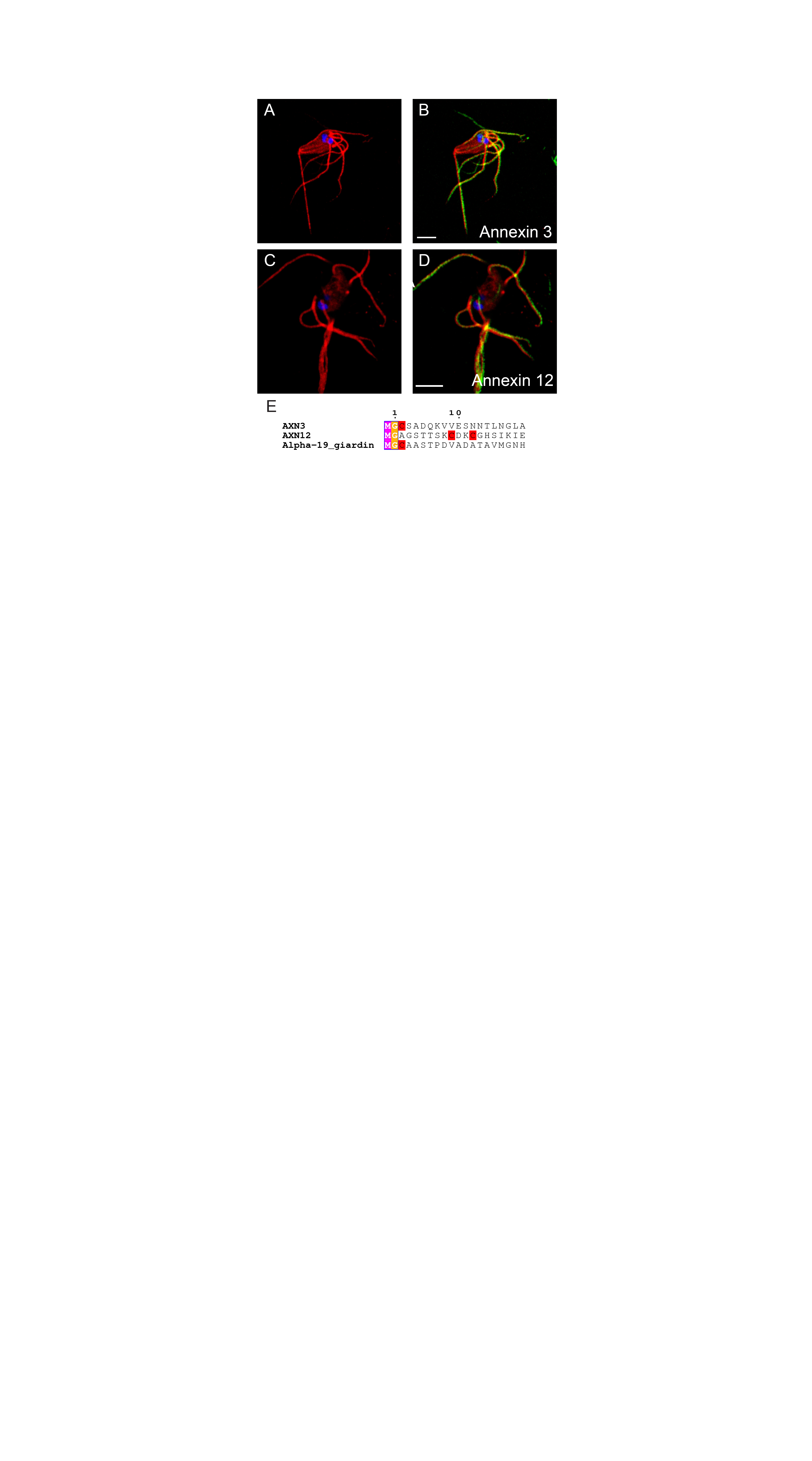


**Figure S7**

Supplement: Figure S7 [file sph002162052sf7.docx]

**
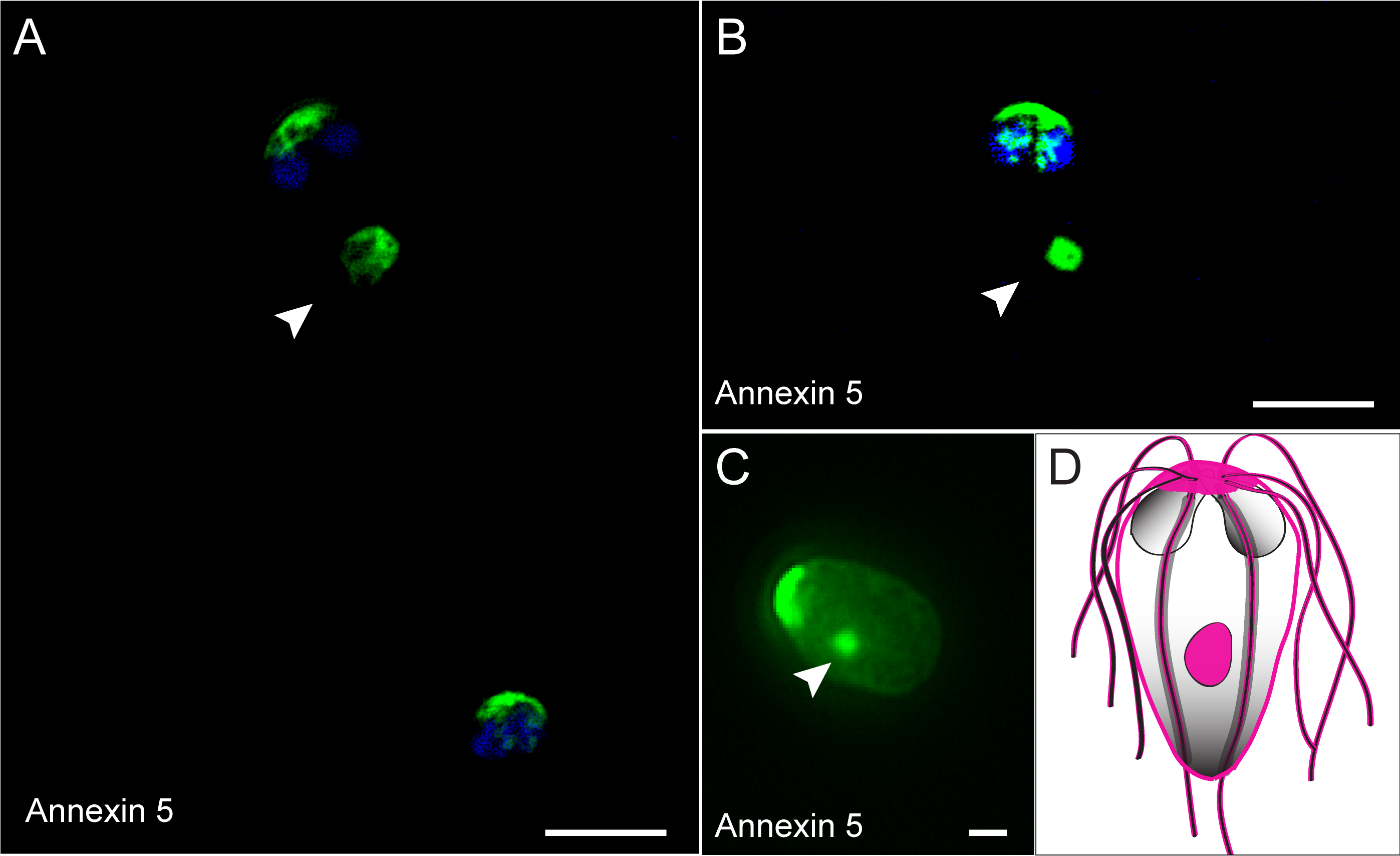
**

**Figure S8**

Supplement: Figure S8 [file sph002162052sf8.docx]

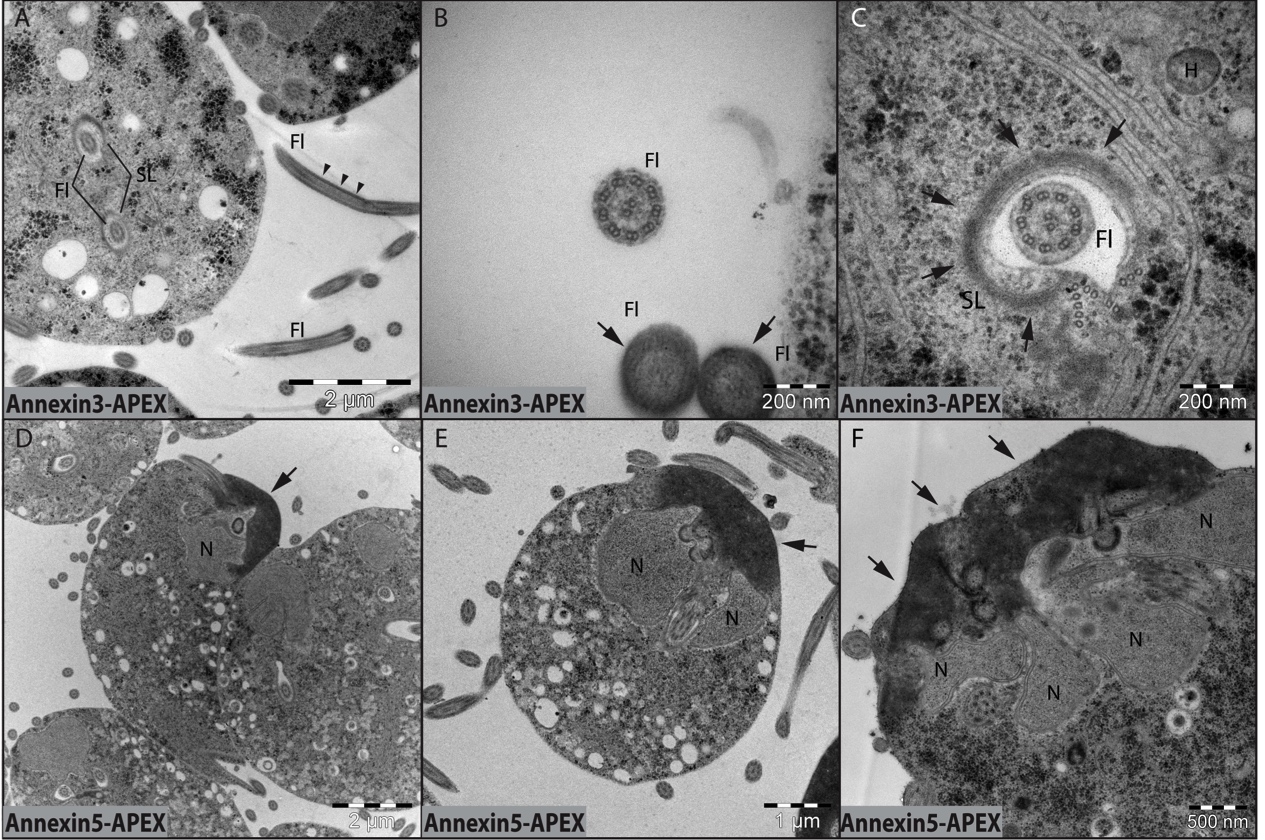


**Figure S9**

Supplement: Figure S9 [file sph002162052sf9.docx]
