# Supplementary material for: Comparative Cell Biology and Evolution of Annexins in Diplomonads
Source: mSphere. 2016 Mar 23;1(2):e00032-15. doi: 10.1128/mSphere.00032-15 (PMC4863580; doi:10.1128/mSphere.00032-15)
Supplement: Table S1 [file sph002162052st10.docx]

| **Primer name** | **Sequence (5’- 3’)** |
| --- | --- |
| *Primers for cloning of Annexins* | |
| Annexin1-F | CCCACGCGTGCTACTTAGATCTATTCAGCCAATCTTAC |
| Annexin1-R | CCCGCGGCCGCTGGGCCTTCCAAATTGCCAATAATG |
| Annexin2-F | CCCACGCGTCCATTTAGCTTTACGATCTGCCAT |
| Annexin2-R | CCCGCGGCCGCTTTGCGTCCCATATTAAAAGGAGG |
| Annexin3-F | CCCACGCGTGAATGACGACAGCGCGTATTAAAG |
| Annexin3-R | CCCGCGGCCGCAAGCCCCAGAAAGCCAAGAC |
| Annexin4-F | CCCACGCGTGTCAATTATTATTCACTAGCCAAATTTG |
| Annexin4-R | CCCGCGGCCGCTCTTATCCCCAGAGGGCAAG |
| Annexin5-F | CCCACGCGTGGAGGAATATGTAGTGGTATGCTTTGTG |
| Annexin5-R | CCCGCGGCCGCAAGCCCCAAAGAGCAAGAACGAG |
| Annexin6-F | CCCACGCGTGCACAGGTGTTCCTTTACTCTTTAC |
| Annexin6-R | CCCGCGGCCGCTTCAACCCCCATACTGCTAAAATG |
| Annexin7-F | CCCACGCGTGCTATCCGGCGTTGTATTGAACG |
| Annexin7-R | CCCGCGGCCGCGATTTAAGATTCCACAGCTCTAATATTCC |
| Annexin8-F | CCCACGCGTGCAAAGGATGGCGGTTAAGAATG |
| Annexin8-R | CCCGCGGCCGCTTCCAAAGAGTAATAATAGTATCAGATATATC |
| Annexin9-F | CCCACGCGTAGCAGGATGCCATGTAGAGC |
| Annexin9-R | CCCGCGGCCGCCTCAGGCCCCATACGGCGAGGAC |
| Annexin10-F | CCCACGCGTGTGAAATTCAAATCGAAAGATTTCAGG |
| Annexin10-R | CCCGCGGCCGCCCACAGCATTTCTTGAAAAGGTTACC |
| Annexin11-F | CCCACGCGTCTGCCAGGATAGATGGTTCATTTG |
| Annexin11-R | CCCGCGGCCGCTTAAATCCAAAAAAACTTAAAACAGCATTTTAG |
| Annexin12-F | CCCACGCGTGGATATACAAACATTTTGCATAACCCATAAG |
| Annexin12-R | CCCGCGGCCGCAGGCCCCAGAAAGCTAAAATAGC |
| Annexin13-F | CCCACGCGTCAGCGTTAGTGGTGCAAAGTCATG |
| Annexin13-R | CCCGCGGCCGCATCTGCAAAATAGAACAGATTACATCAACC |
| Annexin14-F | CCCACGCGTGTGGGTTTTTGGAGGGTTTTGGGGCAAC |
| Annexin14-R | CCCGCGGCCGCAAAATGGTAATAGCTTTTTTAAAGATCCCACAGC |
|  |  |
| *Primers for construction of pSpiro-PAC-APEX-V5 vector* | |
| APEX-F | GCGGCCGCCCGGCGGATCAGGCTCTGG |
| APEX-V5-R | GGGCCCCGTAGAATCGAGACCGAGGAGAGGGTTAGGGATAGGCTTACCTGCCTCGGCGAATCCCAGTTC |
|  |  |

**Table S1**
